# Evaluation of rupture risk of pediatric intracranial aneurysm using high-resolution magnetic resonance imaging techniques: a case report

**DOI:** 10.1186/s12883-026-05040-y

**Published:** 2026-08-03

**Authors:** Xin Feng, Lele Dai, Wenxin Chen, Yuxin Li, Yiming Bi, Zhuohua Wen, Shixing Su, Xin Zhang, Xifeng Li, Chuanzhi Duan

**Affiliations:** 1 Neurosurgery Center, Department of Cerebrovascular Surgery, Engineering Research Center of Diagnostic and Therapeutic Technology and Devices for Cerebrovascular Diseases, Ministry of Education, Zhujiang Hospital, Southern Medical University, Guangzhou, Guangdong 510280 China; 2 Guangdong Provincial Key Laboratory on Brain Function Repair and Regeneration, Guangzhou, Guangdong 510280 China; 3 Department of Radiology, Zhujiang Hospital, Southern Medical University, 253 Gongye Middle Avenue, Haizhu District, Guangzhou, Guangdong 510282 China

**Keywords:** Pediatric intracranial aneurysm, Subarachnoid hemorrhage, High-resolution magnetic resonance imaging, 3D vessel wall imaging, Wall enhancement.

## Abstract

This case report details the association between vessel wall enhancement and the “weak points” of a giant pediatric intracranial aneurysm. A 14-year-old female presented her headache at admission and suffered from an acute aneurysm rupture before scheduled surgery. Examination of the preoperative vessel wall imaging revealed three high-intensity segments on the aneurysm wall on 3D visual analysis, indicating a high rupture risk. This unique case highlights the potential of high-resolution magnetic resonance imaging in identifying high-risk aneurysms and offers valuable evidence for clinical decision-making in a rare ruptured pediatric case.

## Background

Pediatric intracerebral hemorrhage (pICH) is rare and carries potentially permanently disastrous psychosocial and medical consequences [2]. In the pediatric population, intracranial aneurysm (IA) is a rare disease, comprising less than 5% of all IAs [1]. However, once a child-onset IA ruptures, the mortality rate can exceed 10% [5]. The natural history of pediatric intracranial aneurysm (pIA) differs from cases in the adult population, with a higher occurrence in the anterior circulation and a male predominance [4, 11]. Therefore, the strategies for assessing rupture risk are specific and cannot be based only on evidence from the adult population.

The common initial symptoms of pIA include dizziness, headaches, and seizures. However, these clinical symptoms are usually non-specific, making diagnosis challenging. Despite the increasing utilization of high-resolution medical imaging techniques in recent years, there is still a lack of evidence developing definitive recommendations for assessing rupture risk in pIA due to the limited number of clinical cases.

Our case is that of a 14-year-old female without a history of trauma or significant past morbidities. The patient presented to the pediatric neurosurgery outpatient clinic after a gradual-onset headache persisting for two months and was subsequently diagnosed with a giant left vertebral artery dissection aneurysm. Cerebrovascular-related diseases are a field where high-resolution magnetic resonance imaging (HR-MRI) techniques, such as aneurysm wall enhancement (AWE) imaging, are frequently applied. However, studies evaluating the effectiveness of HR-MRI for the assessment of the risk of pIA rupture have been limited to date.

## Case presentation

The patient’s headaches began two months before her presentation and progressively worsened over the last two weeks. She felt a throbbing pain in her head after a physical education class, prompting her parents to take her to a local hospital for a CT scan, which revealed a space-occupying lesion in the brain stem. Initially, the frequency of her headache attacks was 2–3 times a day, intensifying during physical activity. Two weeks prior to admission, there was an escalation in the frequency and severity of her headache episodes, occurring even at rest and typically lasting approximately 2 to 3 min. She also experienced occasional dizziness when straining during bowel movements but did not report instability on her feet. The patient denied limb weakness, numbness of limbs, hypomnesia, nausea, vomiting, visual deficit, diplopia, or blurred vision. In the outpatient clinic, her headache did not occur, despite two weeks of insomnia. At admission, her heart rate was slightly elevated, but other vital signs were normal: temperature 36.8 ℃, pulse rate 113 bpm, respiration 21 times per minute, and blood pressure 124/85 mm Hg. A neurological examination did not reveal any abnormal signs. The patient’s motor and sensory neuron functions are entirely normal. Auscultation of her lungs was clear. Cardiac examinations did not show any irregular heart rhythm or murmurs.

**Fig. 1:**
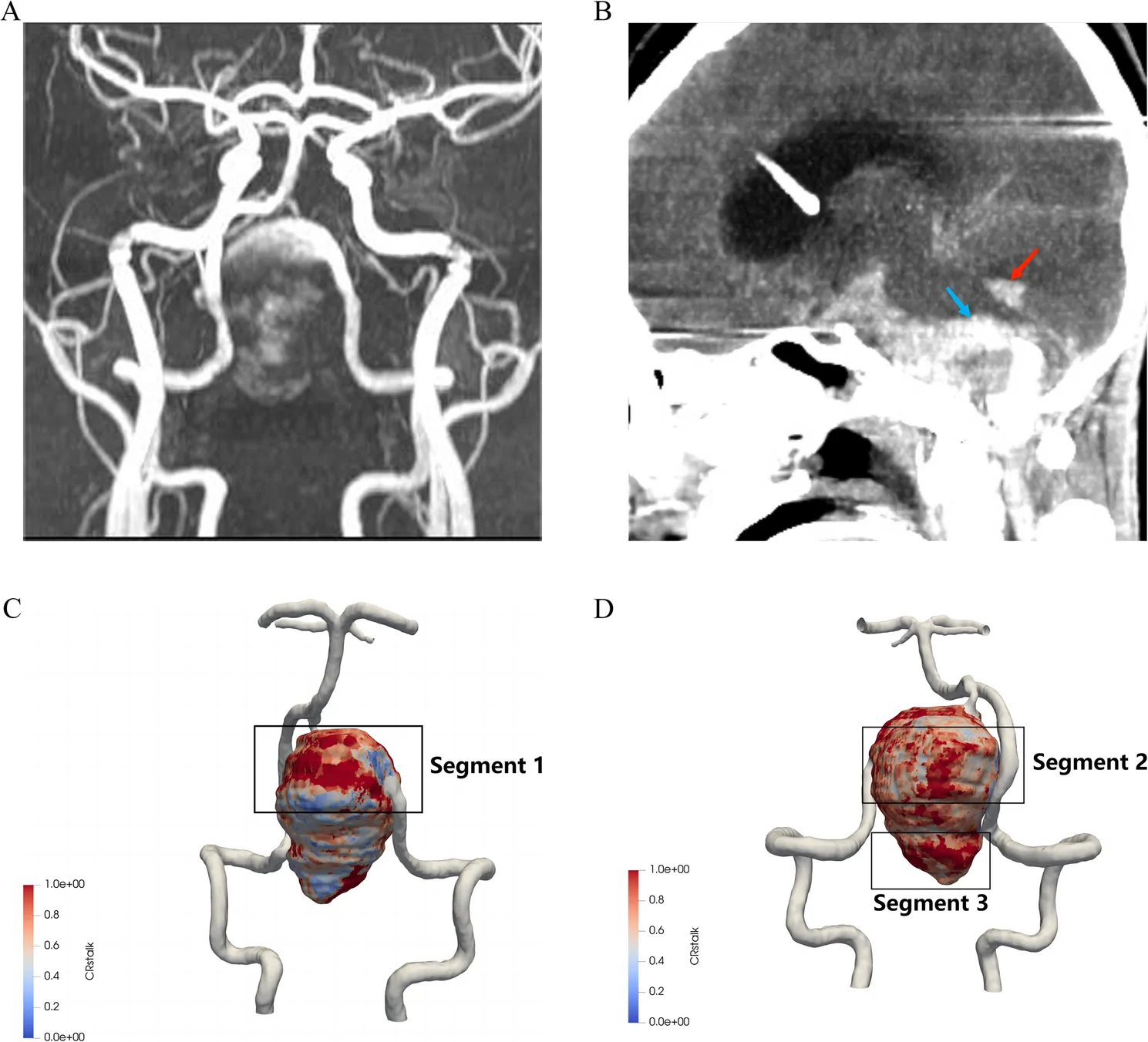
High-resolution vessel wall imaging (HR-VWI) and 3D quantitative analysis of wall enhancement in a dissecting aneurysm. **A** MR angiography illustrating the overall morphology of the cerebral vasculature. **B** Sagittal 3D T1-weighted black-blood MRI showing pronounced focal enhancement (arrows) and significantly thickened wall (up to 1.5 mm). **C** and **D** 3D pseudo-color mapping of the aneurysm wall enhancement (AWE). The color scale (CRstalk) reflects the signal intensity ratio normalized against the pituitary stalk. Segmental mapping (Segments 1–3) intuitively demonstrates the spatial distribution of pronounced focal enhancement (red areas), indicating localized structural vulnerabilities and a high risk of rupture

High-resolution vessel wall imaging (HR-VWI) was performed using a 3.0-T MRI scanner equipped with a 3D T1-weighted black-blood sequence. The specific scanning parameters were as follows: repetition time (TR) = 800 ms, echo time (TE) = 20 ms, field of view (FOV) = 180 × 180 mm, and an isotropic slice thickness of 0.5 mm. To accurately quantify aneurysm wall enhancement (AWE) and avoid physiological variations, the signal intensity of the aneurysm wall was normalized against that of the pituitary stalk. Furthermore, the signal intensity ratio of the aneurysm wall to the corpus callosum was calculated to be 1.25, indicating visible focal enhancement relative to the reference tissue. Previous studies using signal intensity ratios have generally considered higher aneurysm wall-to-reference tissue ratios to reflect more prominent aneurysm wall enhancement. However, this finding should be interpreted cautiously because standardized pediatric thresholds for aneurysm wall enhancement have not yet been established. Morphological measurements demonstrated a marked discrepancy in vessel wall thickness: the non-ruptured segment of the vertebral artery measured 0.4 mm, whereas the maximum wall thickness at the ruptured site of the dissecting aneurysm was significantly thickened to 1.5 mm. These localized structural vulnerabilities and intense enhancement patterns strongly suggest an elevated risk of rupture in this pediatric case, highlighting the need for tailored neuroimaging evaluations. (Fig. 1).

The parents of the patient decided to receive endovascular treatment to prevent occurrence of hemorrhagic stroke. Unfortunately, the aneurysm ruptured two hours before the scheduled endovascular treatment, and despite immediate intervention, the patient died two weeks after the procedure as a result of a catastrophic natural history of aneurysmal subarachnoid hemorrhage. Significantly, the postoperative CT images confirmed that the bleeding lesion corresponds to segment 1, consistent with the preoperative assessment.

### Discussion and conclusions

The impact of HR-MRI utilization across neurosurgery is an area of developing interest, especially for cerebrovascular disease. A previous study has suggested the predictive capabilities of AWE for aneurysm rupture risk [7]. In this adolescent case, three main advantages of normalizing AWE by the SI of the pituitary stalk were identified: (1) suggesting that AWE is also a high-risk factor for rare pIA rupture; (2) providing a quantitative technique to visualize AWE in three dimensions; and (3) complementing existing methods for predicting aneurysm rupture risk, such as morphological assessments and finite element analysis (FEA).

In pediatric patients, IA tend to be large, giant and non-saccular [10]. For giant IAs, prominent blood stagnation effect inside aneurysm could result in hyperintense signal which might be mistaken for gadolinium enhancement. Therefore, a superficial assessment of AWE based on emission computed image may introduce inevitable bias. Hemodynamic studies based on FEA have consistently served as a significant tool for assessing aneurysm rupture risk. However, as the boundary conditions of this method assume rigid wall structures, its calculations may not entirely align with clinical practice [9]. Additionally, in children, limitations in the slicing algorithm relying on stereolithography model are amplified by the overall small diameter of the adolescent vasculature. A particular benefit of HR-MR is its ability to noninvasively detect “weak spots” in the aneurysm wall, whereas other examinations such as CT angiography (CTA) and digital subtraction angiography (DSA) require children to be exposed to X-rays, leading to potential safety risks of radioactive damage.

Indeed, children typically account less than 2% of patients with aneurysmal hemorrhage according to published series [6]. Non-specific symptoms may be the only clinical sign of aneurysm rupture, underscoring the need for heightened awareness among surgeons when dealing with pediatric patients. Aneurysmal subarachnoid hemorrhage in particular can have various triggers, such as fever, exercise, infection, inflammation, and extreme fluctuations in systemic blood pressure caused even by a severe cough [3, 8]. This rare case illustrates that clinicians can evaluate the risk of pIA rupture through the HR-MRI and AWE, emphasizing the importance of early identification and timely intervention to prevent devastating pICH. Despite these findings, our study has several limitations that must be acknowledged. First, as a single case report, the evidence level is inherently limited, and the findings cannot be broadly generalized. Second, there is a lack of large-scale, prospective pediatric cohort data to validate the exact threshold of AWE in predicting rupture risk for pediatric populations. Third, due to retrospective data constraints, we were unable to perform a patient-specific hemodynamic assessment (such as computational fluid dynamics) to cross-validate our imaging findings with local flow patterns. Future multicenter studies with larger sample sizes and comprehensive hemodynamic evaluations are necessary to verify our observations.

## Data Availability

No datasets were generated or analysed during the current study.

## Abbreviations

pIAs: Pediatric intracranial aneurysms
HR-MRI: High-resolution magnetic resonance imaging
pICH: Pediatric intracerebral hemorrhage
AWE: Aneurysm wall enhancement
SI: Signal intensity
FEA: Finite element analysis
CTA: CT angiography
DSA: Digital subtraction angiography
